# Vitamin E-added Highly Cross-Linked Polyethylene Decreases the Risk of Osteolysis in an In Vivo Arthroplasty Model

**DOI:** 10.7759/cureus.34955

**Published:** 2023-02-14

**Authors:** Celaleddin Bildik, Hamit Çağlayan Kahraman, Baransel Saygı

**Affiliations:** 1 Orthopaedics and Traumatology, Ataşehir Florence Nightingale Hospital, Istanbul, TUR; 2 Orthopaedics and Traumatology, Fatih Sultan Mehmet Training and Research Hospital, İstanbul, TUR; 3 Orthopaedics and Traumatology, Özel Pendik Bölge Hospital, Istanbul, TUR

**Keywords:** animal study, sprague-dawley rats, vitamin e, antioxidant, polyethylene, aseptic loosening, arthroplasty

## Abstract

Introduction

Aseptic loosening is one of the most important complications of arthroplasty surgery. It is known that immune response against particles plays role in the pathogenesis of aseptic loosening. Polyethylene (PE) has an important place in these particles. There are limited in vivo studies examining aseptic loosening caused by PE residues.

Objective

The aim of the present study is to evaluate the aseptic loosening created by highly cross-linked PE (HXLPE) and vitamin E-added PE particles in an in vivo knee prosthesis model.

Materials and methods

Thirty-nine male Sprague-Dawley rats, which were randomized into three groups, were included in the study. After surgical exposure of knee joints of rats, femoral intramedullary canals were drilled and instilled with isolated saline solution and saline solution that contained standard PE or vitamin E-added PE particles according to their groups. Afterwards, a titanium implant was placed on the femoral articular surface of each animal. Rats received intraarticular injections weekly of the same solution, which was initially instilled into their femoral canal. The rats were sacrificed at the end of the third week and then underwent radiological and histopathological evaluations.

Result

In histopathological evaluation, periprosthetic membrane formation, inflammatory cell change, and cellular damage of cartilage and bone tissue around the implant were assessed. There was a statistically lesser amount of cellular damage and periprosthetic membrane formation in the vitamin-E/HXLPE group compared to the HXLPE group (p=0.04, p=0.001). No significant difference was found between the PE groups with respect to inflammatory cells (p=0.715).

Conclusions

HXLPE caused more significant osteolysis compared to VE-HXLPE. Antioxidants in PE could provide a reduction in osteolysis and aseptic loosening.

## Introduction

Arthroplasty, when performed with the proper indication and method, alleviates arthritis-related joint discomfort and promotes functional recovery. Thereby, it increases patients' quality of life significantly. However, it might not reach the intended painless joint and functional result, so the necessity of revision surgery can occur. Arthroplasty revision, which causes more medical expenses, has higher complication rates and lesser satisfactory results. Over one million total joint replacement surgery are considered to be performed worldwide every year, and the number of revision surgeries is expected to increase in the coming years [1-3]. Aseptic loosening, instability, infection, and septic loosening are among the most important causes of revision surgery after total joint arthroplasty [4,5]. Extra measures must be taken to prevent these complications from occurring. When the cost burden of revision surgery is considered, the importance of studies on the avoidance of causes has come to the forefront.

Aseptic loosening is the topmost reason for revision surgery [3-5]. The pathogenesis of aseptic loosening and osteolysis may occur depending on the characteristics of the patient and implant [6-8]. The development of new biomaterials for increasing the survival of prostheses is very important in order to reduce the need for revision surgery and additional medical treatment in terms of the burden on the patient and the national economy.

Total joint replacement revision surgeries are operations with high cost and complication rates. It is, thus, very important to delay and decrease the rate of aseptic loosening, which is one of the main reasons for revision surgery. One of the main reasons for the failure of arthroplasty is the production of debris particles due to the articulation interface, which causes the reaction of the foreign body to occur. Particulate residues consisting of metals, polyethylene (PE), ceramic and bone cement have been shown to trigger a biological response in joint tissues [9-11]. PE is one of the most commonly used insert materials, especially in knee arthroplasty. Thanks to the low-cost development of PE materials used in arthroplasty, aseptic loosening and revision arthroplasties, which can lead to serious complications and large expenditures, can be reduced.

The aim of our study is to investigate the effect of vitamin E-added highly cross-linked PE (HXLPE) on the formation of aseptic loosening in an in vivo knee prosthesis model.

## Materials and methods

Yeditepe University Experimental Animal Studies Council approved this experimental study on July 14, 2014 (approval number: 408). All procedures performed in studies involving animals were in accordance with the ethical standards of the institution and practice at which the studies were conducted. This study was carried out at Yeditepe University Experimental Research Center and Yeditepe University Faculty of Basic Medical Sciences Laboratory, Istanbul, Türkiye.

Aseptic loosening in arthroplasty surgery was assessed by using different types of PE particles on 39 Sprague-Dawley male rats. All of the rats used in this study were seven months old, with an average weight of 380 g (range 320-450 g) and the same genetic characteristics. Nutrition of animals was fulfilled with standard rat diet and tap water before and after surgery. Rats were under controlled temperature (21 ± 1 °C) and lighting conditions (12 hours light/dark cycle). Rats were randomly divided into three groups. Anesthesia of all rats was provided by intraperitoneal administration of a mixture of 50 mg/kg ketamine and 15 mg/kg xylazine. Due to the loss of two rats under anesthesia, 12 animals were included in the first group, 13 in the second group, and 12 in the third group. After surgical exposure of knee joints, femoral intramedullary canals of animals were washed and suctioned. Then canals were instilled with pure saline (SF) (Group 1), saline containing HXLPE particles (Group 2), and saline containing vitamin E-HXLPE (VE-HXLPE) particles (Group 3). Titanium implants were placed in the femoral articular surface after instillation. Then injections into the knee joints of rats were made in weekly intervals with SF in Group 1, SF containing HXLPE particles in Group 2, and SF containing VE-HXLPE particles in Group 3. One researcher performed all surgical procedures and he was blinded from the study and did not know the injection group.

Experimental design and operative technique

**Figure 1:**
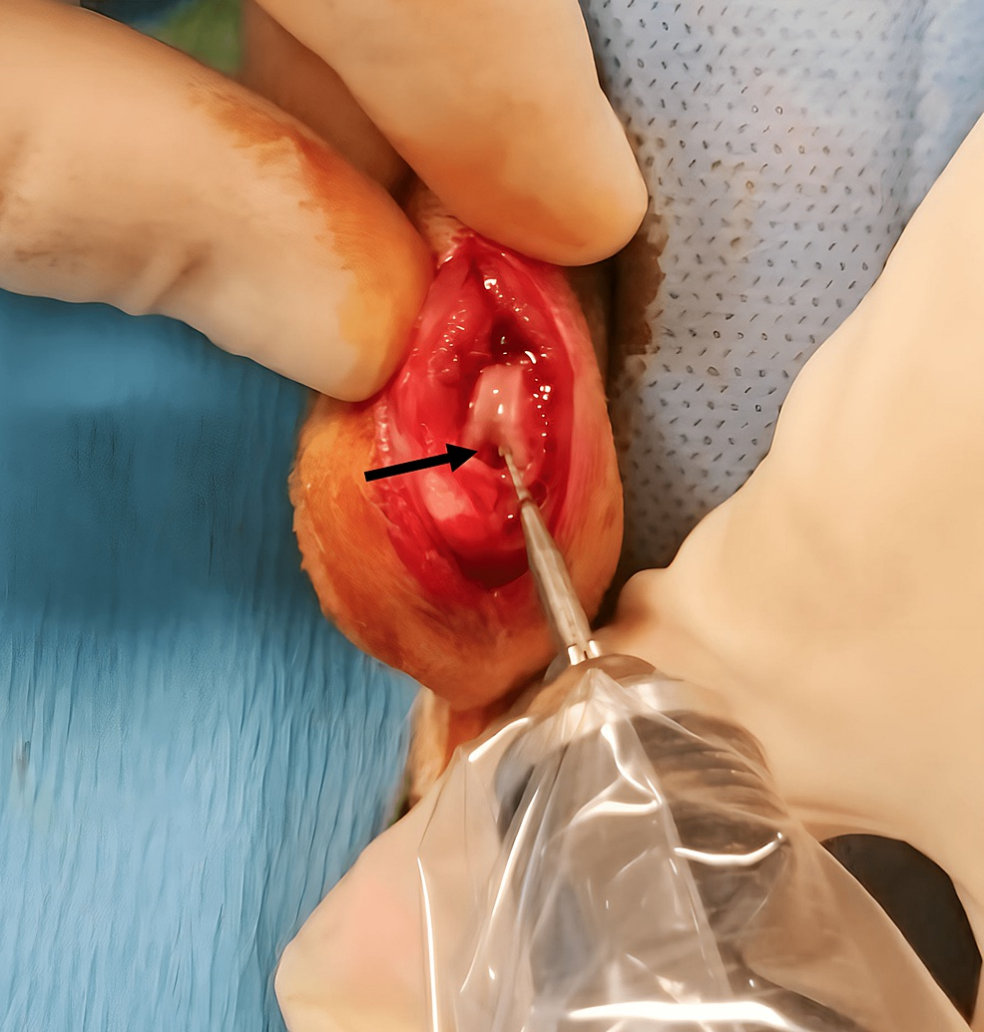
Opening of femoral canal with the help of dentist drill The black arrow indicates the entrance of the femoral canal

The right leg was shaved preoperatively. The skin was cleaned with an iodine solution three times after scrubbing with an iodophor for five minutes. A longitudinal skin incision was made and the knee joint was exposed by medial parapatellar capsulotomy without the use of a tourniquet. A dental drill of 0.8 mm diameter was used to penetrate the cortex of the intercondylar notch of the femur into the medullary canal (Figure 1).

HXLPE particles, VE-HXLPE particles, and titanium implants were prepared in advance. The titanium implant was manufactured by TST Medical Devices, Istanbul, Türkiye, and had a pin length of 9.2 mm, a diameter of 1 mm, and a head diameter of 2.5 mm. PE particles, the average size of which was 3.1 ± 0.7 μm, were obtained from the mold by surface cutting and grinding path [12]. The titanium knee implants were sterilized in an autoclave. PE particles were kept in 100% alcohol for an hour for sterilization [13]. For injections, 5 mg HXLPE particles into 15 cc saline and 5 mg of VE-HXLPE particles into 15 cc saline were stated separately in order to prepare mixtures according to their groups [14]. These prepared suspensions were used for the intramedullary canal instillation and for weekly knee joint injections.

**Figure 2:**
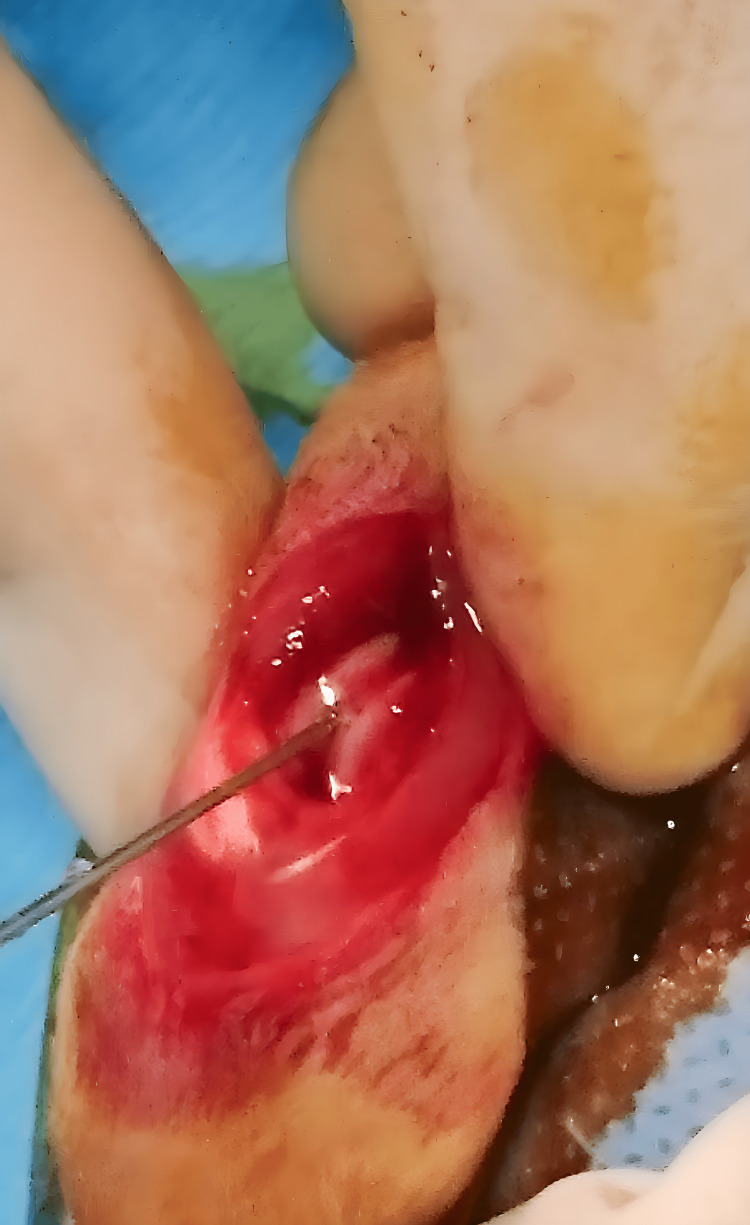
Femoral intramedullary canal injection

After irrigation and suction of the canal, the medullary canal was instilled with 0.1 cc of SF for Group 1, 0.1 cc of HXLPE particle-containing suspension for Group 2, and 0.1 cc VE-HXLPE particle-containing suspension for Group 3 (Figure 2) [15].

**Figure 3:**
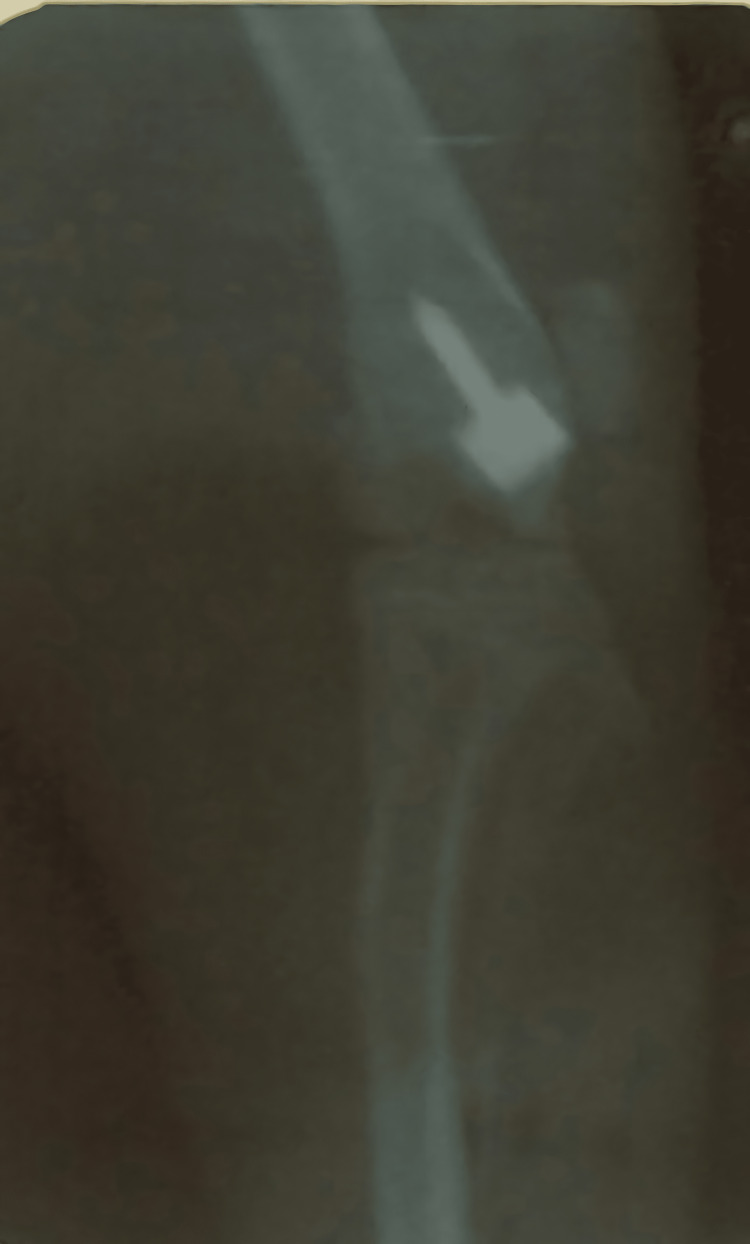
Postoperative X-ray of rat knee

In order to investigate the presence of osteolysis at the intramedullary canal implant-bone interface, the implant designed as a femoral component was placed in the intramedullary canal of the femur as hemiarthroplasty. According to the average weight and size of the rats used in the study, one size implant made of titanium was used. Care was taken not to cause any mechanical restriction in the knee joint. The head portion of the implant, which was on femoral articular surface, was checked for patellar contact and range of motion. The capsule and skin were closed with suture. No movement restriction was applied to the rats. A conventional radiograph was taken for each animal in order to check the position of the implant with a periapical X-ray device (New Life Radiology SRL, Turin, Italy) (Figure 3).

Feeding and watering were continued in standard protocols for rats. Wound care of the rats was performed every day postoperatively. There was no evidence of infection macroscopically. At the end of the first week postoperatively, two rats, which were selected from each group using the block randomization method, were sacrificed in order to have baseline histopathological parameters after radiographs of their knee joint were taken of their knee joints. The same procedure was performed at the end of the second week. At the end of the third postoperative week, the remaining rats of each group were sacrificed for histopathologic and radiographic evaluation. After sacrification by using carbon dioxide ventilation, both legs of the rats were cut from the proximal femur and mid-diaphyseal tibia and were placed in 4% formaldehyde for histopathologic examination. All histopathological data obtained from sacrificed rats in the first, second, and third weeks were compared within and between groups. The histopathologist was not informed about the group of specimens.

Histopathological evaluation 

Tissues, which were pre-fixed with 4% formaldehyde, were decalcified in Biodec-R (Bio Optica Milano SPA, Milan, Italy). Then they were dehydrated by passing through ascending series of alcohol (70%, 90%, 96%, 100%) and embedded in paraffin. Sections of 4 µm thickness were prepared and stained with hematoxylin and eosin. Stained sections were examined for periprosthetic membrane formation and inflammatory cells in the tissue around the implant; cellular damage on cartilage and bone around the implant and photographed under a light microscope (Leica DM 500; Leica Microsystems GmbH, Wetzlar, Germany). These three criteria were evaluated as 0 = none, 1 = low, 2 = moderate, and 3 = severe [14,15].

Statistical analysis of histopathological data was performed by NCSS (Number Cruncher Statistical System) 2007 (NCSS, LLC. Kaysville, Utah, United States). The minimum sample size required to detect a significant difference using this test should be at least 36 considering type I error (alfa) of 0.05, power (1-beta) of 0.8, and effect size of 0.55. Descriptive statistical methods were used to evaluate the data (mean, SD, median). Kruskal-Wallis test for comparisons between groups and Dunn's multiple comparison test for comparisons of subgroups were used as well. A p-value of < 0.05 was considered to be significant. 

## Results

At about the first postoperative week, gnawing behavior was observed in two rats, one from Group 1 and the other from Group 3. Opening portions of the wound were small and re-sutured. Except for these, no problem was seen in the wound healing of rats after the surgical procedure. In follow-up, no sign of infection or difference in eating, behavior, and movement was observed in any animal, including the two re-sutured ones. The rats were also evaluated after being sacrificed; there were observed healed surgical wound sites and intact joint capsules.

Radiographic evaluation

The knee radiographs taken after the sacrification of rats were compared with their first postoperative radiographs. Radiological assessments were performed by an orthopedic surgeon. A radiological sign of aseptic loosening, which is a radiolucent line around the implant, was not observed in plain X-rays of all three groups.

Histopathological evaluation

**Figure 4:**
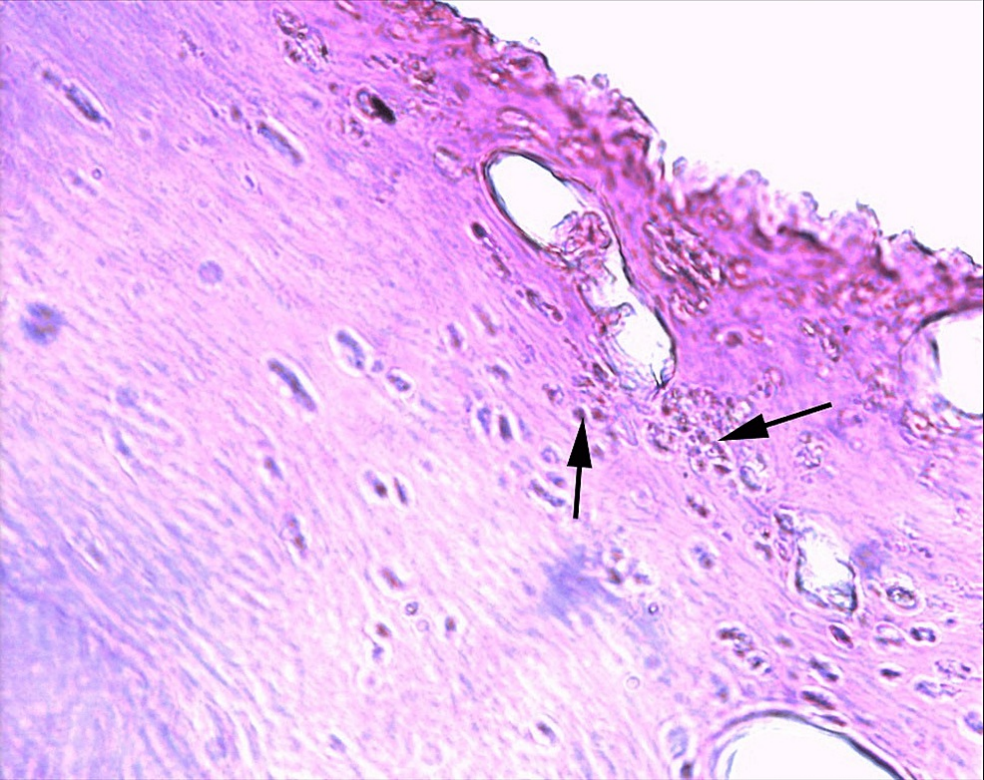
Inflammatory cells migrating around the prosthesis

There was a statistically significant difference observed in the amount of inflammatory cells, periprosthetic membrane formation, and cellular damage among the three groups in the third week (p=0,0001). An increase in the number of inflammatory cells in Group 1 was found to be significantly lower than in Group 2 and Group 3 (p=0,001). There was no statistically significant difference between Group 2 and Group 3 in the number of inflammatory cells (p = 0.715) (Tables 1, 2) (Figure 4).

**Table 1:** Weekly presentation of inflammatory cells in groups Group 1: pure saline group; Group 2: HXLPE group; Group 3: vitamin E-HXLPE group IQR: interquartile range; HXLPE: highly cross-linked polyethylene

Inflammation (Inflammatory cell)		Group 1	Group 2	Group 3	p-value
1^st^ postoperative week	Mean±SD	0.5±0.71	2±0	1.5±0.71	0.167
	Median (IQR)	0.5 (0-0.75)	2 (1.5-1.5)	1,5 (0.75-2)	
2^nd^ postoperative week	Mean±SD	1±0	2±0	2±0	0.082
	Median (IQR)	1 (0.75-0.75)	2 (1.5-1.5)	2 (1.5-2)	
3^rd^ postoperative week	Mean±SD	1.13±0.35	2.33±0.5	2.25±0.46	0.0001
	Median (IQR)	1 (1-1)	2 (2-3)	2 (2-2.75)	

**Table 2:** Statistical comparison of inflammatory cell values between groups Group 1: pure saline group; Group 2: HXLPE group; Group 3: vitamin E-HXLPE group HXLPE: highly cross-linked polyethylene

Dunn's Multiple Comparison Test	1^st ^postoperative week (p-value)	2^nd^ postoperative week (p-value)	3^rd^ postoperative week (p-value)
Group 1/Group 2	0.102	0.083	0.001
Group 1/Group 3	0.221	0.083	0.001
Group 2 /Group 3	0.317	0.999	0.715

**Figure 5:**
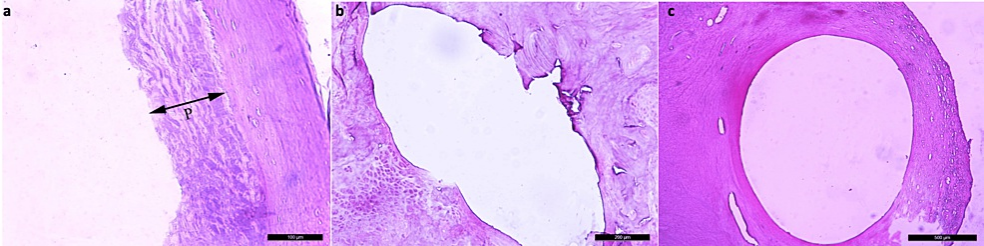
Excessive periprosthetic membrane formation around the prosthesis

The periprosthetic membrane thickness in Group 2 was found to be significantly thicker than in Group 1 (p=0.0001) and Group 3 (p=0.001). No statistically significant difference was observed between Group 1 and Group 3 in terms of periprosthetic membrane thickness (p=0.317) (Tables 3, 4) (Figure 5).

**Table 3:** Weekly mean±SD and median values of periprosthetic membrane values Group 1: pure saline group; Group 2: HXLPE group; Group 3: vitamin E-HXLPE group IQR: interquartile range; HXLPE: highly cross-linked polyethylene

Periprosthetic membrane		Group 1	Group 2	Group 3	p-value
1^st^ postoperative week	Mean±SD	1±0	2±0	1±0	0.082
	Median (IQR)	1 (0.75-0.75)	2 (1.5-1.5)	1 (0.75-1.25)	
2^nd^ postoperative week	Mean±SD	1±0	2.5±0.71	1±0	0.091
	Median (IQR)	1 (0.75-0.75)	2.5 (1.5-2.25)	1 (0.75-1.25)	
3^rd^ postoperative week	Mean±SD	1±0	2.44±0.53	1.13±0.35	0.0001
	Median (IQR)	1 (1-1)	2 (2-3)	1 (1-1)	

**Table 4:** Statistical comparison of periprosthetic membrane values among the groups Group 1: pure saline group; Group 2: HXLPE group; Group 3: vitamin E-HXLPE group HXLPE: highly cross-linked polyethylene

Dunn's Multiple Comparison Test	1^st^ postoperative week (p-value)	2^nd ^postoperative week (p-value)	3^rd^ postoperative week (p-value)
Group 1/Group 2	0.083	0.102	0.0001
Group 1 /Group 3	0.999	0.999	0.317
Group 2/Group 3	0.083	0.102	0.001

**Figure 6:**
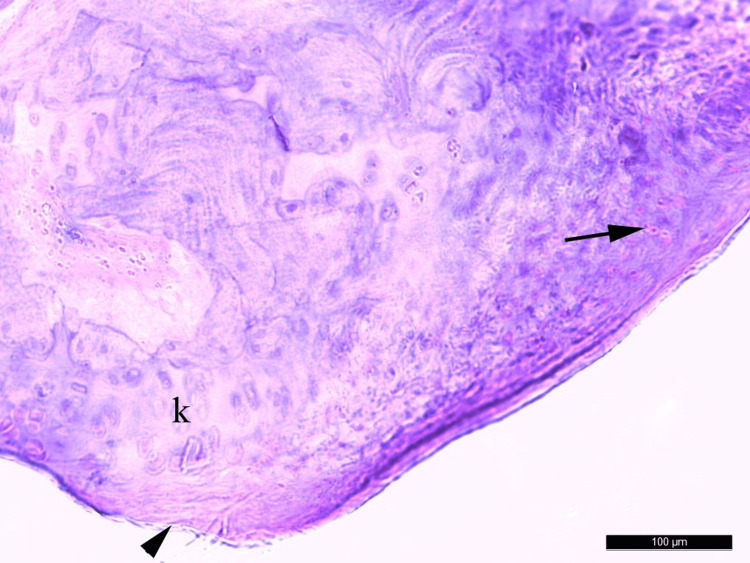
Eosinophilic inflammatory cells and synovial membrane damage in the articular surface cartilaginous tissue around the prosthesis

**Figure 7:**
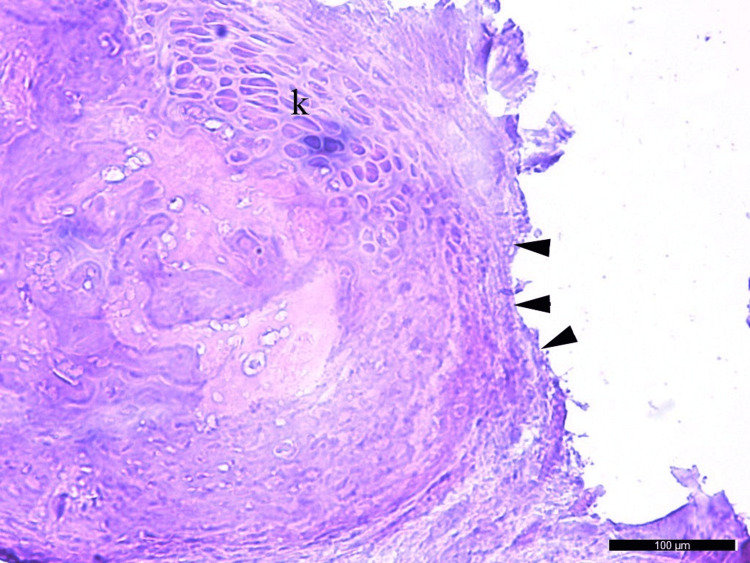
Synovial membrane damage

The cellular damage in Group 1 was significantly lower than in Group 2 (p=0.001) and Group 3 (p=0.008). There was significantly more cellular damage in Group 2 compared to Group 3 (p=0,004). (Tables 5, 6) (Figures 6, 7).

**Table 5:** Weekly mean±SD and median values of cellular damage values Group 1: pure saline group; Group 2: HXLPE group; Group 3: vitamin E-HXLPE group IQR: interquartile range; HXLPE: highly cross-linked polyethylene

Cellular damage		Group 1	Group 2	Group 3	p-value
1^st^ postoperative week	Mean±SD	0±0	2±0	1±0	0.082
	Median (IQR)	0 (0-0)	2 (1.5-1.5)	1 (0.75-1.25)	
2^nd^ postoperative week	Mean±SD	0.5±0.71	2±0	1±0	0.115
	Median (IQR)	0.5 (0-0.75)	2 (1.5-1.5)	1 (0.75-1.25)	
3^rd^ postoperative week	Mean±SD	0.38±0.74	2.56±0.53	1.5±0.54	0.0001
	Median (IQR)	0 (0-0.75)	3 (2-3)	1.5 (1-2)	

**Table 6:** Statistical comparison of the cellular damage values between groups Group 1: pure saline group; Group 2: HXLPE group; Group 3: vitamin E-HXLPE group HXLPE: highly cross-linked polyethylene

Dunn's Multiple Comparison Test	1^st^ postoperative week (p-value)	2^nd^ postoperative week (p-value)	3^rd ^postoperative week (p-value)
Group 1/Group 2	0.083	0.102	0.001
Group 1/Group 3	0.083	0.317	0.008
Group 2/Group 3	0.083	0.083	0.004

## Discussion

In the present study, we compared HXLPE and VE-HXLPE particles for osteolysis in an experimental arthroplasty model to see if there is a difference between them. It was demonstrated and confirmed by an animal model that the addition of vitamin E to HXLPE reduced the incidence of osteolysis and relaxation, due to the lower inflammatory response produced. The reason these materials were chosen for the present investigation is that they were the most commonly used PE nowadays. In the current study, tissue samples taken from surrounding titanium metal implants were compared in terms of periprosthetic membrane thickness, the number of inflammatory cells, and the amount of cellular damage. Statistically significant differences were observed in the number of inflammatory cells, periprosthetic membrane formation, and cellular damage. 

In the current study, it was demonstrated that the VE-HXLPE particles caused a significantly lower amount of cellular damage and periprosthetic membrane thickness compared to HXLPE. In the assessment of inflammatory cell number, although no statistically significant difference was observed, HXLPE particles were found to cause the production of more inflammatory cells compared to VE-HXLPE and SF.

Bone resorption and aseptic loosening around the prosthesis is the most common long-term complication of total joint replacement surgery. Due to the negative effects of loosening on the patient's daily life, pain and mobility problems increase. The cause and pathophysiology of aseptic loosening have yet to be deduced, especially when considering the high cost and complication rates of revision surgeries. A balance between new bone formation and osteolysis around the implant determines the loosening. Diagnosis of loosening is usually made by demonstration of osteolysis, due to PE wear, as well as implant migration in severe cases around the implant, radiographically and with its clinical correlations [9]. 

It has been shown that particle debris, from prosthetic components due to mechanical reasons, creates periprosthetic inflammation and causes osteolysis [16]. Loosening in arthroplasty surgery has been shown to occur in this way [17,18]. It has been also demonstrated in studies that bone resorption occurred due to factors interleukin (IL)-1, IL-6, tumor necrosis factor (TNF), prostaglandin E2 (PGE2), and collagenase, which had been produced by immune responses against particles [19-22]. The rates of these mediators were observed to be quite high in tissue samples taken around loose knee and hip arthroplasty implants [23-25].

The most important causative factor in the pathogenesis of aseptic loosening is the inflammatory immune response, which develops due to mechanical debris from components of the prosthesis [4,26]. After the emergence of the disadvantages of metal-on-metal prostheses and the description of aseptic lymphocytic vasculitis-associated lesions (ALVAL), their use has decreased considerably. The most common prosthetic options today are still metal on PE, ceramic on PE, or ceramic on ceramic. In addition, the PE used has evolved from ultra-high molecular weight PE (UHMWPE) to HXLPE for better wear properties producing fewer particles and hence longer life. For assessment of the response against particles, histopathological analysis had been done to look for periprosthetic membrane thickness, cell damage, and inflammation amount and also for observation of osteoclastic activity [26].

Biological responses against any one or combination of biomaterials from prosthetic components such as PE, Cr-Co, ceramics, and titanium had been studied in animal models [27]. Zhang et al., in their study, injected titanium particles intra-articularly after inserting the titanium implant into the tibia [27]. They then observed the resulting inflammatory responses. Histopathological examination showed that the periprosthetic membrane thickness had increased with titanium particle injection. Matthew Allen et al., in their study on rats, placed ceramic implants on the tibia [14]. After repeated intra-articular injections of PE particles, they observed the rise of chronic inflammatory cells in the periprosthetic tissue.

In the current study, in a similar way, we applied a titanium arthroplasty model implant to rat femur. Before insertion of implants, intramedullary canals of rats were instilled with SF, saline containing HXLPE particles, or saline containing VE-HXLPE particles according to their group. Following weekly intraarticular injections after the surgery, the impact of the particles on the arthroplasty model was compared. Except for the present study, there is no study in the literature evaluating the aseptic loosening created by HXLPE and VE-HXLPE particles in an in vivo prosthesis model. 

Huang et al., in their experimental study, investigated the responses to different polymer particles on the mouse calvarium [26]. In that study, the degree of osteoclastic activity and inflammation had been observed of UHMWPE, HXLPE, and VE-HXLPE particles. Osteoblastic activity and inflammation against vitamin E-containing PE was found to be less than the others.

In Van Der Vis et al.’s study, responses to different particles were observed after repeated injections of them into the knee of rats [28]. Each group containing UHMWPE, high-density PE, Cr-Co, zirconium, titanium, and latex particles in similar size and density had been researched and evaluated. They did not find any difference in the synovial tissue of the control and particle-injected groups. However, the thickness of the synovial tissue was found to be a little bit higher in high-density PE and UHMWPE groups. The authors commented that particles alone were insufficient to initiate bone resorption. In the second part of the same study, after opening the femoral medulla, the canal was washed with particulate solutions and closed with a plug of bone cement. Similar histopathological results were obtained from both parts of the study.

The subject was investigated, the results were evaluated as statistically significant, and it was thought that it would shed light for future studies. According to studies, particles were thought to increase bone resorption and also have negative effects on new bone formation [13]. There are distinguished opinions about metal debris particles, which are from commonly used materials in orthopedic surgery, that they inhibit metabolic activity and have cytotoxic properties on osteoblastic cells [29,30].

Studies on particle-induced inflammation and osteolysis are very important to reduce inflammation and loosening in order to improve prosthesis longevity and survival.

New studies on the immune response against arthroplasty biomaterials and cytokines released would lead to the production of safer biomaterials, which might reduce the occurrence of osteolysis. As a result, the need for revision surgery caused by aseptic loosening could be reduced considerably.

Limitations of the study

First, the type of implant used in the study was not a total replacement of a joint. Second, electron microscopic images could provide a clearer view. In future studies, besides light microscopic examination, electron microscopy examination of arthroplasty models containing total joint replacement specially produced for animal models can be investigated. 

## Conclusions

Vitamin E can reduce the formation of free radicals, probably due to its antioxidant effect. Consequently, a reduction of osteolysis might be obtained. In the present study, the vitamin E-embedded PE was found to be more secure as an insert material in terms of aseptic loosening formation. HXLPE caused more significant osteolysis compared to VE-HXLPE. Antioxidants in PE could provide a reduction in osteolysis and aseptic loosening.

## References

[1] Teeny SM, York SC, Mesko JW, Rea RE (2003). Long-term follow-up care recommendations after total hip and knee arthroplasty: results of the American Association of Hip and Knee Surgeons' member survey. J Arthroplasty.

[2] Kurtz S, Ong K, Lau E, Mowat F, Halpern M (2007). Projections of primary and revision hip and knee arthroplasty in the United States from 2005 to 2030. J Bone Joint Surg Am.

[3] Ren K, Dusad A, Zhang Y, Wang D (2013). Therapeutic intervention for wear debris-induced aseptic implant loosening. Acta Pharmaceutica Sinica B.

[4] Hallab NJ, Jacobs JJ (2009). Biologic effects of implant debris. Bull NYU Hosp Jt Dis.

[5] Holt G, Murnaghan C, Reilly J, Meek RM (2007). The biology of aseptic osteolysis. Clin Orthop Relat Res.

[6] Kadoya Y, Revell PA, Kobayashi A, al-Saffar N, Scott G, Freeman MA (1997). Wear particulate species and bone loss in failed total joint arthroplasties. Clin Orthop Relat Res.

[7] Shanbhag AS, Jacobs JJ, Black J, Galante JO, Glant TT (1994). Macrophage/particle interactions: effect of size, composition and surface area. J Biomed Mater Res.

[8] Buly RL, Huo MH, Salvati E, Brien W, Bansal M (1992). Titanium wear debris in failed cemented total hip arthroplasty. An analysis of 71 cases. J Arthroplasty.

[9] Jiang Y, Jia T, Wooley PH, Yang SY (2013). Current research in the pathogenesis of aseptic implant loosening associated with particulate wear debris. Acta Orthop Belg.

[10] Wooley PH, Petersen S, Song Z, Nasser S (1997). Cellular immune responses to orthopaedic implant materials following cemented total joint replacement. J Orthop Res.

[11] Tanoğlu O, Say F, Yücens M, Alemdaroğlu KB, İltar S, Aydoğan NH (2020). Titanium alloy intramedullary nails and plates affect serum metal ion levels within the fracture healing period. Biol Trace Elem Res.

[12] Fang HW, Hsu SM, Sengers JV (2003). Chapter 7 surface texture design and generation of narrowly distributed particles. NIST Special Publication: Ultra-High Molecular Weight Polyethylene Wear Particle Effects on Bioactivity.

[13] Taki N, Tatro JM, Nalepka JL, Togawa D, Goldberg VM, Rimnac CM, Greenfield EM (2005). Polyethylene and titanium particles induce osteolysis by similar, lymphocyte-independent, mechanisms. J Orthop Res.

[14] Allen M, Brett F, Millett P, Rushton N (1996). The effects of particulate polyethylene at a weight-bearing bone-implant interface. A study in rats. J Bone Joint Surg Br.

[15] Ortiz SG, Ma T, Regula D, Smith RL, Goodman SB (2008). Continuous intramedullary polymer particle infusion using a murine femoral explant model. J Biomed Mater Res B Appl Biomater.

[16] Saleh KJ, Thongtrangan I, Schwarz EM (2004). Osteolysis: medical and surgical approaches. Clin Orthop Relat Res.

[17] Yang SY, Yu H, Gong W, Wu B, Mayton L, Costello R, Wooley PH (2007). Murine model of prosthesis failure for the long-term study of aseptic loosening. J Orthop Res.

[18] Yang SY, Wu B, Mayton L, Mukherjee P, Robbins PD, Evans CH, Wooley PH (2004). Protective effects of IL-1Ra or vIL-10 gene transfer on a murine model of wear debris-induced osteolysis. Gene Ther.

[19] Glant TT, Jacobs JJ, Molnár G, Shanbhag AS, Valyon M, Galante JO (1993). Bone resorption activity of particulate-stimulated macrophages. J Bone Miner Res.

[20] Murray DW, Rushton N (1990). Macrophages stimulate bone resorption when they phagocytose particles. J Bone Joint Surg Br.

[21] Maloney WJ, Smith RL, Castro F, Schurman DJ (1993). Fibroblast response to metallic debris in vitro: enzyme induction, cell proliferation and toxicity. J Bone Joint Surg Am.

[22] Haynes DR, Rogers SD, Hay S, Pearcy MJ, Howie DW (1993). The differences in toxicity and release of bone-resorbing mediators induced by titanium and cobalt- chromium-alloy wear particles. J Bone Joint Surg Am.

[23] Goldring SR, Schiller AL, Roelke M, Rourke CM, O’Neil DA, Harris WH (1983). The synovial-like membrane at the bone-cement interface in loose total hip replacements and its proposed role in bone lysis. J Bone Joint Surg.

[24] Appel AM, Sowder WG, Siverhus SW, Hopson CN, Herman JH (1990). Prosthesis-associated pseudomembrane-induced bone resorption. Br J Rheumatol.

[25] Chiba J, Schwendeman LJ, Booth RE, Crossett LS, Rubash HE (1994). A biochemical, histologic and immunohistologic analysis of membranes obtained from failed cemented and cementless total knee arthroplasty. Clin Orthop.

[26] Huang CH, Lu YC, Chang TK (2016). In vivo biological response to highly cross-linked and vitamin e-doped polyethylene--a particle-induced osteolysis animal study. J Biomed Mater Res B Appl Biomater.

[27] Zhang T, Yu H, Gong W, Zhang L, Jia T, Wooley PH, Yang SY (2009). The effect of osteoprotegerin gene modification on wear debris-induced osteolysis in a murine model of knee prosthesis failure. Biomaterials.

[28] Van Der Vis HM, Marti RK, Tigchelaar W, Schüller HM, Van Noorden CJ (1997). Benign cellular responses in rats to different wear particles in intra-articular and intramedullary environments. J Bone Joint Surg Br.

[29] Rae T (1981). The toxicity of metals used in orthopaedic prostheses. An experimental study using cultured human synovial fibroblasts. J Bone Joint Surg Br.

[30] Allen MJ, Millett PJ, Myer BJ, Rushton N (1995). The effects of particulate cobalt, chromium and cobalt-chrome alloy on human osteoblast-like cells in vitro. Trans Europ Orthop Res Soc.

